# Ultrasound-Assisted Liposuction (UAL) Arm Contouring in Non-Post-Bariatric Patients: No Rush for Brachioplasty

**DOI:** 10.1007/s00266-022-03070-8

**Published:** 2022-08-30

**Authors:** Mohammed Hassan El-Fahar, Ahmed Hassan El-Gharbawi

**Affiliations:** 1 Plastic and Reconstructive Surgery Department, Faculty of Medicine, Mansoura University, 60 El Gomhoria St, Mansoura, DK 35516 Egypt; 2 Consultant of Plastic and Reconstructive Surgery, Hihya Plastic and Burn Center, Hihya, Egypt

## Abstract

**Background:**

A witnessed rise in patients’ requests for arm contouring reflects the parallel public’s pursuit of slimmed bodies and rapid advancement in weight reduction methods. Brachioplasty with its known complications is still the traditional method of management, but nearly all patients feel worried about the length and appearance of the scar and seek other non-excisional alternatives. The authors wanted to share their experience in arm contouring in non-post-bariatric patients using ultrasound-assisted liposuction (UAL).

**Patients and Methods:**

Over 16 month period, 28 female patients complaining of arm lipodystrophy (classes IIA, IIB, III) underwent UAL contouring under general anesthesia. Preoperative and postoperative mid-arm circumferences were measured and recorded. Outcome evaluation was done by the complication incidence, patient satisfaction survey, and independent surgeon evaluation of patients' photographs.

**Results:**

There were no complications in the study group. The outcome evaluation survey has shown high patient satisfaction. The outcome survey demonstrated that 85.71% of the patients were very satisfied, while 14.29 % were satisfied with the procedure and all of them recommend the procedure to others. On the other hand, the independent surgeon evaluation showed that 92.86% of the results were excellent and 7.14 % were very good.

**Conclusion:**

Our work has shown how versatile is the UAL in contouring a wide spectrum of arm lipodystrophy stages in non-post-bariatric patients and presents a non-excisional alternative for arm aesthetic refinements without a rush for brachioplasty with its unpleasant complications.

**Level of Evidence IV:**

This journal requires that authors assign a level of evidence to each article. For a full description of these Evidence-Based Medicine ratings, please refer to the Table of Contents or the online Instructions to Authors www.springer.com/00266.

## Introduction

The first brachioplasty was introduced by Correa-Iturraspe and Fernandez in the 1950s [1]. The aesthetic contouring of the arms without skin incisions becomes a demanding goal to achieve youthfulness. The greatest obstacle is the relative skin recoil nature to the surrounding anatomical structures in the arm and the axilla [2]. Understanding the concept of adherence helps minimize contour deformities in suction-assisted liposuction [3]. Brachioplasty has become the most effective way to reshape the arm in patients with lipodystrophy and extensive skin redundancy [4]. The procedure itself is always associated with some unpleasant compilation and uneventful outcome that necessitated the evolution of multiple techniques to improve the aesthetic outcome and decrease the complications [5]. Teimourian and Malekzadeh reported that the optimum outcome depends on modifying any procedure to match the anatomical configuration of the arm. The upper extremity rejuvenation surgeon should possess knowledge of a myriad of techniques to enhance the result for the patient. Regardless of the procedure used, scars from brachioplasty are often wide or hypertrophic and frequently require revision [6]. Unfortunately, many patients become hesitant to seek liposuction only because they have been told that brachioplasty is the only option to achieve beautiful arms**.** Those patients are concerned about visible scars and complications [4]. Gilliland and Lyos suggested liposuction to correct arm lipodystrophy; however, their work was limited to patients with early stages of lipodystrophy [7]. The introduction of ultrasonic-assisted liposuction achieved less blood loss and preserved adipocyte-derived stem cells that might play a role in quiescent skin retraction [8]. A literature review, done by Shermak A. in 2014, had shown different approaches and classification systems that vary according to patient presentation and the author's opinion and most of them are forms of brachioplasty that may include liposuction in single or two stages, which reveals a lack of consensus. He emphasized that the most annoying factor in brachioplasty is patient consciousness about the presence and the location of the scar especially when it is complicated with hypertrophy [9]. El-Khatib classified brachioplasty according to the amount of adipose tissue deposit and the degree of ptosis in which stage I has a minimal amount of adipose tissue without skin ptosis; stage IIA has a moderate amount of adipose tissue with skin ptosis less than 5 cm (grade I ptosis); stage IIB has a large amount of adipose tissue with skin ptosis of 5–10 cm (grade II ptosis); stage III has a large amount of adipose tissue with skin ptosis greater than 10 cm (grade III ptosis), and stage IV has a minimal amount of adipose tissue with grade III ptosis [10]. In this study, the authors aimed to share their experience in arm contouring in non-post-bariatric patients (stage I–IV according to El-Khatib’s classification) [10], exploring how versatile is UAL in contouring a wide spectrum of arm lipodystrophy while avoiding scar problems and increasing patient satisfaction.

## Patients and Methods

The authors conducted a retrospective study on 28 female patients who underwent arm contouring between July 2020 and October 2021. The data have been collected from patients who were admitted to our university hospital and the authors’ private practices. Our inclusion criteria were adult female patients between 18 and 60 years old with no previous bariatric procedure, while our exclusion criteria were BMI above 40, patients with unrealistic expectations, and any medical morbidity e.g., diabetes or smoking. Full history taking was conducted emphasizing weight stability, current BMI, cleared medical comorbidities, no active psychological issues, and reasonable patient expectations. With the approval of our institutional review board committee under No.R.22.03.1650.R1, written consent of the patient photography and the surgical procedure is obtained. The authors mandated all the ethical considerations regarding the operation procedure and possible complications. The rates of revision for recurrence or complications were discussed with the patients during the consultation. Our study ensures confidentiality regarding all the data that will be taken from the participants. All research participants are free to be enrolled in the study after explaining all the options for management according to their situation. Also, any patient can withdraw at any time from the research project. A thorough arm examination was done in the standing position with the arm 90° abducted and externally rotated and the elbow 90° flexed (victory position). This method allows the surgeon to evaluate the anatomical structure below the bicipital groove, assess the degree of lipodystrophy, and check skin quality, dermal thickness, amount of subcutaneous fat, and presence or absence of striae.

**Fig. 1:**
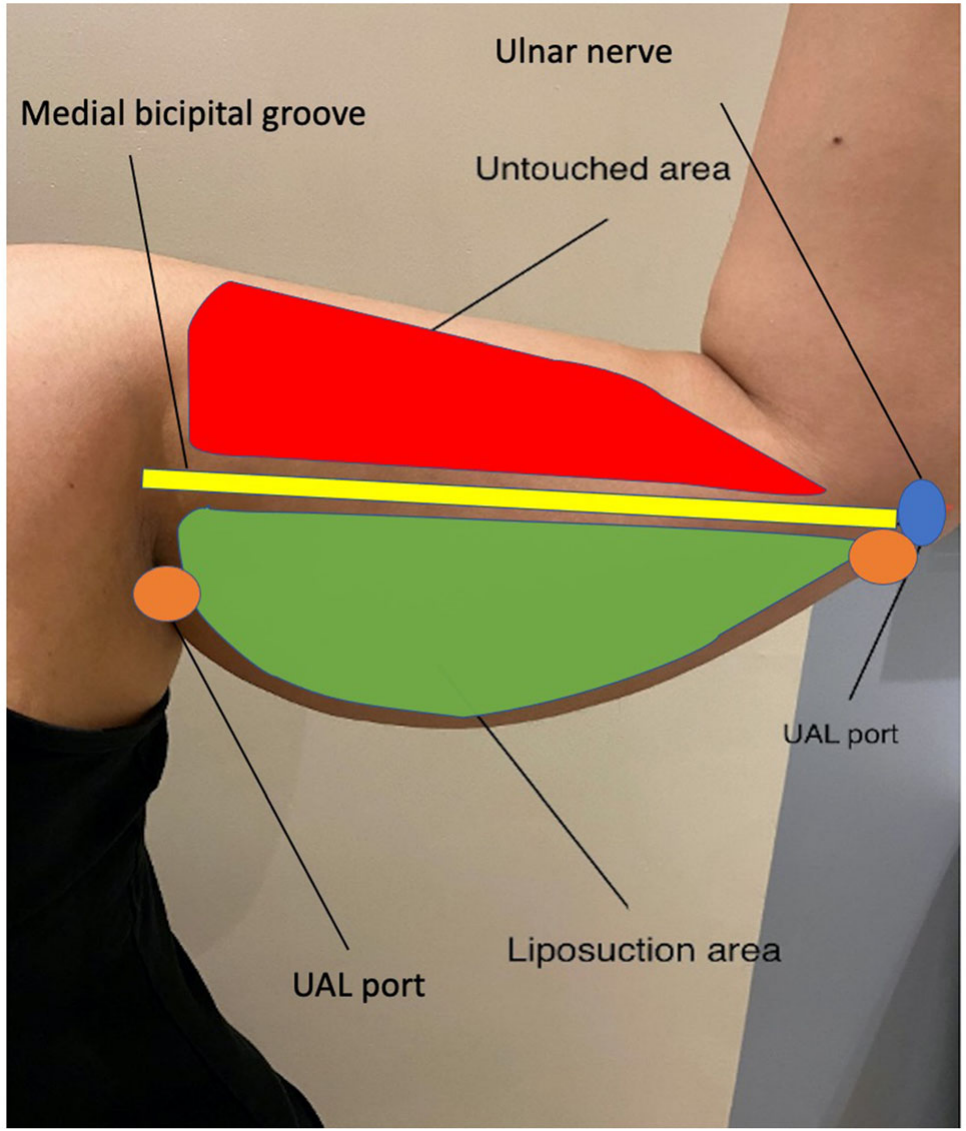
Preoperative photograph showing the zone of liposuction in the lower part of the arm below the bicipital groove (green), the untouched upper region in the form arm (red), the bicipital groove (yellow), the superficial part of the ulnar nerve behind medial epicondyle (blue), and the port of liposuction cannula over the distal part of triceps tendon far away from the ulnar nerve (orange)

According to EL-Khatib’s classification of arm lipodystrophy, class IIA, class IIB, and class III lipodystrophy only were included in our work. Routine preoperative laboratory investigations were done for all cases. Preoperative mid-arm circumference was measured and recorded from all patients. Marking was done, while the patient is in the same position mentioned above starting with a line corresponding to the bicipital groove connecting the anterior axillary fold to the medial epicondyle anteriorly and another from the posterior axillary fold to the lateral epicondyle along with the interval between the biceps and triceps muscles just lateral to deltoid fat pad posteriorly. The author targeted the region inferior to these markings. The location of the ulnar nerve behind the medial epicondyle is marked. Two incision points for the liposuction cannula were placed posterior to the course of the ulnar nerve and behind the posterior axillary line in the axilla. Those two points were away from important structures in the medial bicipital groove, ulnar nerve, and axillary content (Fig. 1). VTE prophylaxis (leg stockings) and 1 gm third-generation cephalosporin 1 h before surgery were done for all cases. All cases were done under general anesthesia in a supine position with the arms abducted 90° on the table. The arms are then prepped and draped, and through 2 small stabs: one just above the medial epicondyle anteriorly and the other between the olecranon process and the medial epicondyle posteriorly, tumescent fluid infiltration is done using ringer lactate solution and epinephrine (ampoule per 500cc bottle) without adding lidocaine. Then ultrasound energy was applied using V mode, 70% power, and 3 grooved tip probe, for a period that was chosen grossly without sticking to specific protocol but varied according to the tumescent volume, respectively. After that SAL was done using a 4 mm Mercedes tip blunt aspiration cannula under 16 inches of vacuum power targeting both the superficial and deep fat compartments. The stab incisions were not closed. A long-arm compression garment with lipofoam was applied intraoperatively and continued for about 6–8 weeks postoperatively. All patients were discharged on the same day after full recovery and were instructed for OPC follow-up regularly. Mid-arm circumference was measured and recorded for all cases at 2 months postoperatively. The follow-up period for 6 months. Outcome evaluation was done by online patient satisfaction survey at 6 months postoperatively, complications incidence, and independent surgeon appraisal of patient photographs (Table 1).

**Table 1:** Patient satisfaction and 3rd surgeon evaluation survey

Item	Question	Response option
Arm look	How toned your upper arms look?	Very satisfied
		Satisfied
		Unsatisfied
Clothes fit	How your clothes’ fitness and size changes after surgery?	Very satisfied
		Satisfied
		Unsatisfied
Scar upset	How noticeable your scars are?	Very upset
		Upset
		Not at all
	How long your scars are?	Very upset
		Upset
		Not at all
Convalescence period	How long the amount of time it would take to heal and recover?	Very satisfied
		Satisfied
		Unsatisfied
Procedure recommendation	Do you recommend procedure to others?	Yes
		No
Result appraisal by 3rd surgeon	How is your evaluation of the result through the submitted pre and postoperative photographs?	Excellent
		Very good
		Good
		Poor

### Statistical Analysis

Data were described using descriptive statistics where qualitative variables were presented as numbers and percentages, while quantitative variables were presented as mean ± SD. Paired *T* test was used to compare preoperative and postoperative mid-arm circumference. All *P*-value information will be descriptive, and *P* < 0.05 was considered significant using IBM SPSS v. 19 (New York, NY, USA).

## Results

**Fig. 2:**
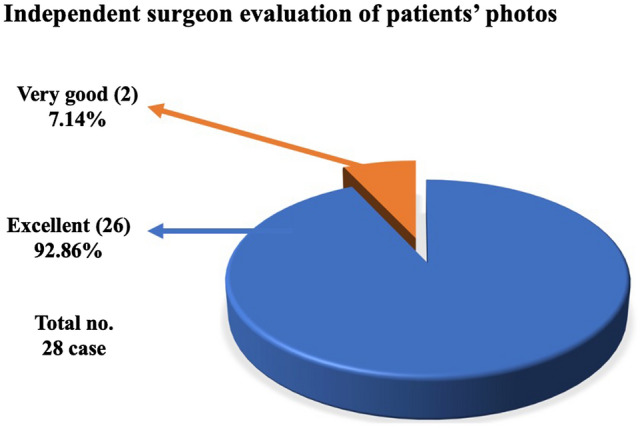
Pie chart showing the independent surgeon evaluation of patient photographs

Our study cohort age ranged between 23 and 42 years with a mean of 33.9±5.7 SD years. Their BMI was between 25 and 35 with a mean of 30.9±5 SD kg/m^2^. Lipodystrophy stages were distributed as follows: 2 cases class IIA, 16 cases class IIB, and 10 cases class III according to El-Khatib’s classification. The ultrasound power time ranged from 5 to 10 min per side with a mean of 7.3±2.2 SD min/side. Our total tumescent volume was 2000± 555 SD cc with a mean total fat aspirate volume of 1646±597 SD cc. The operative time was around 1.3 h and 2.3 h with a mean of 1.75±0.29 SD h. There were no recorded complications. There were no recorded complications (Table 2). While the mean preoperative mid-arm circumference was 43±2.8 SD cm, the mean postoperative mid-arm circumference was 32.9±1.8 SD cm. The mean reduction in mid-arm circumference was 10.1±1.2 SD cm with a 23.5% reduction from the preoperative measurement. A paired *t* test indicated that there is a significantly large difference between pre- and postoperative mid-arm circumference improvement *P* < 0.001 (Table 3). Finally, the outcome survey demonstrated that 85.71% of the patients were very satisfied, while 14.29 % were satisfied with the result (Table 4). On the other hand, the independent surgeon evaluation showed that 92.86% of the results were excellent and 7.14 % were very good (Fig. 2).

**Table 2:** Patients demographics, and outcome data, among the study group (28 patients)

Variable	No. (%)	Range	Mean ± SD
Age(years)		23–42	33.9±5.7
BMI(kg/m^2^)		25–40	30.9±5.6
Lipodystrophy stage:			
Class IIA	2(7)		
Class IIB	16(57)		
Class III	10(36)		
Tumescent volume(cc)		1500–3000	2000±544.3
Fat aspirate volume(cc)		1000–2500	1646±586.1
U/S power time(min/side)		5–10	7.3±2.2
OR time(h)		1.3–2.2	1.75±0.3

**Table 3:** Improvement in mid-arm circumference

Mid-arm circumference	Preoperative (cm)	Postoperative(cm)	Difference in reduction (cm)	Percentage of reduction %	*P* value
Mean	43 ±2.8 SD	32.9 ±1.8 SD	10.1±1.2 SD	23.5%	0.001*

*Paired *T* test, *P*-value 0.05 significance

**Table 4:** Patient satisfaction among the study group

Item	No. (%)
*Arm look*	
Very satisfied	24 (85.71)
Satisfied	4 (14.29)
Unsatisfied	–
*Clothes fit & size change*	
Very satisfied	28(100)
Satisfied	–
Unsatisfied	–
*Scar length & visibility*	
Very upset	–
Upset	–
Not at all	28(100)
*Smoothness of convalescence period*	
Very satisfied	28(100)
Satisfied	–
Unsatisfied	–
*Do you recommend procedure to others?*	
Yes	28(100)
No	–

## Discussion

**Fig. 3:**
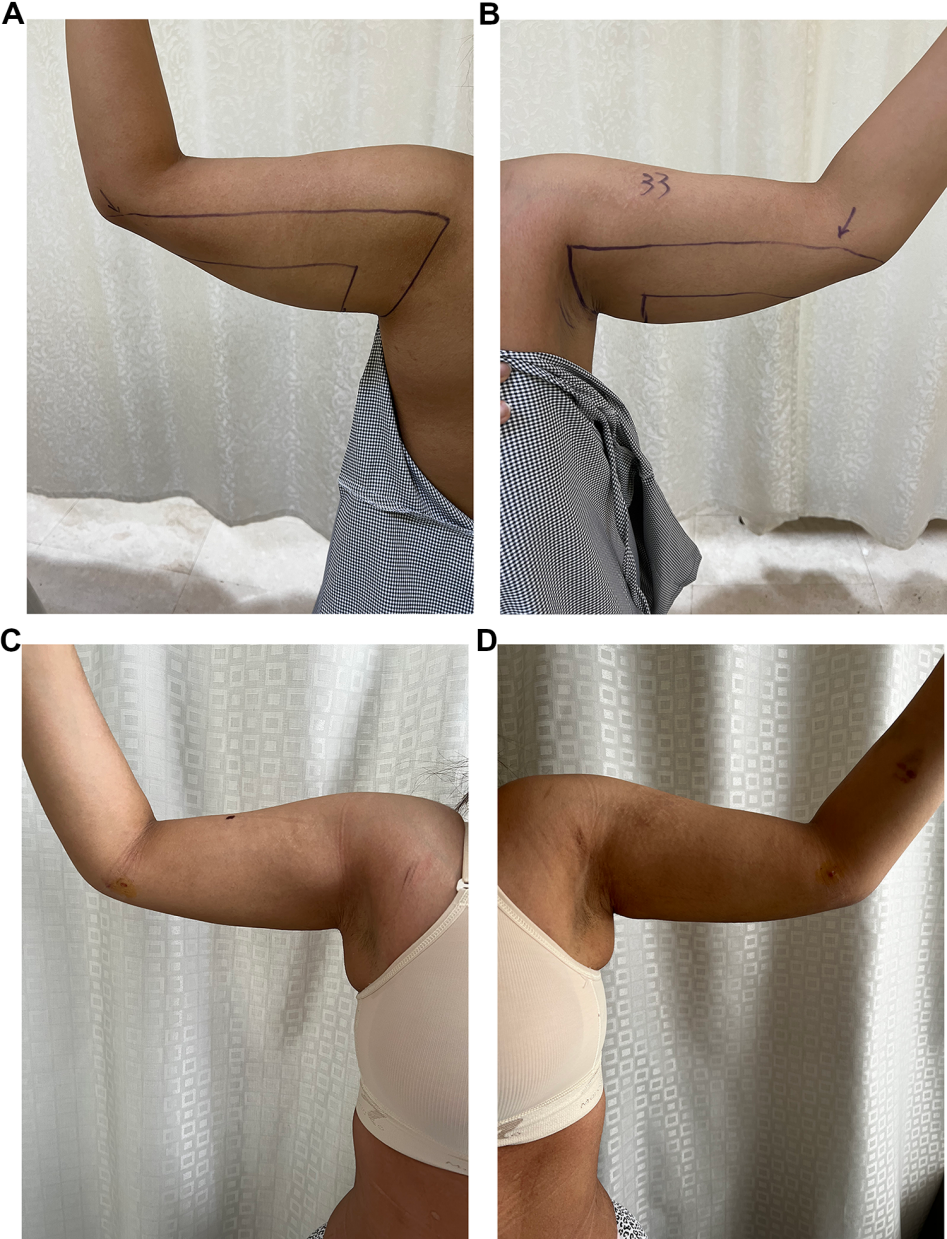
Thirty-five-yo female with class IIA arm lipodystrophy underwent UAL. **A** Right arm perioperative, **B** Left arm perioperative, **C** Right arm 3 month postoperative follow-up, **D** Left arm 3 month postoperative follow-up

**Fig. 4:**
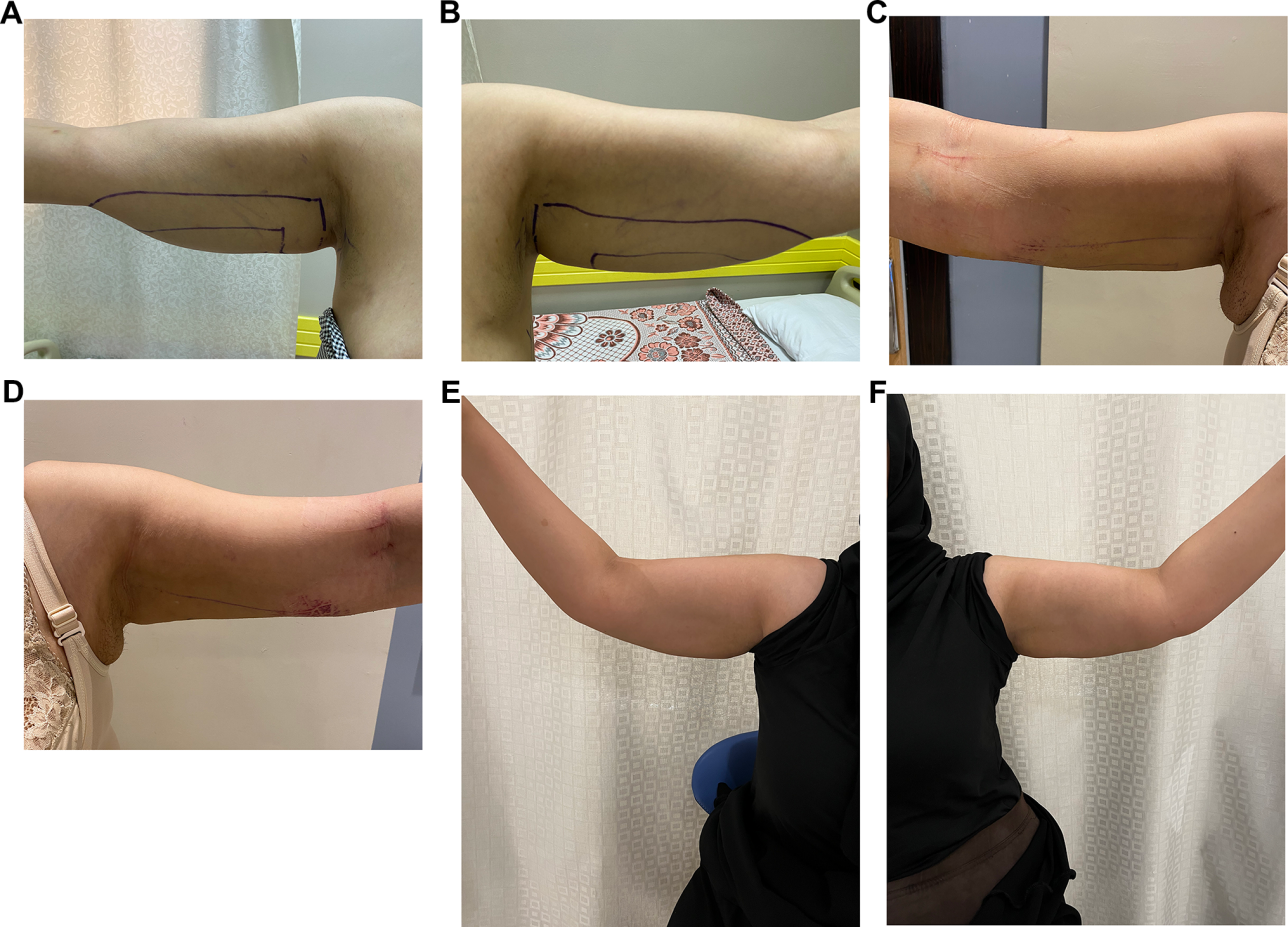
Twenty-eight-yo female with class IIA arm lipodystrophy underwent UAL. **A** Right arm perioperative, **B** Left arm perioperative, **C** Right arm 2 week postoperative follow-up, **D** Left arm 2 week postoperative follow-up, **E** Right arm 1 year postoperative follow-up, **F** Left arm 1 year postoperative follow-up

**Fig. 5:**
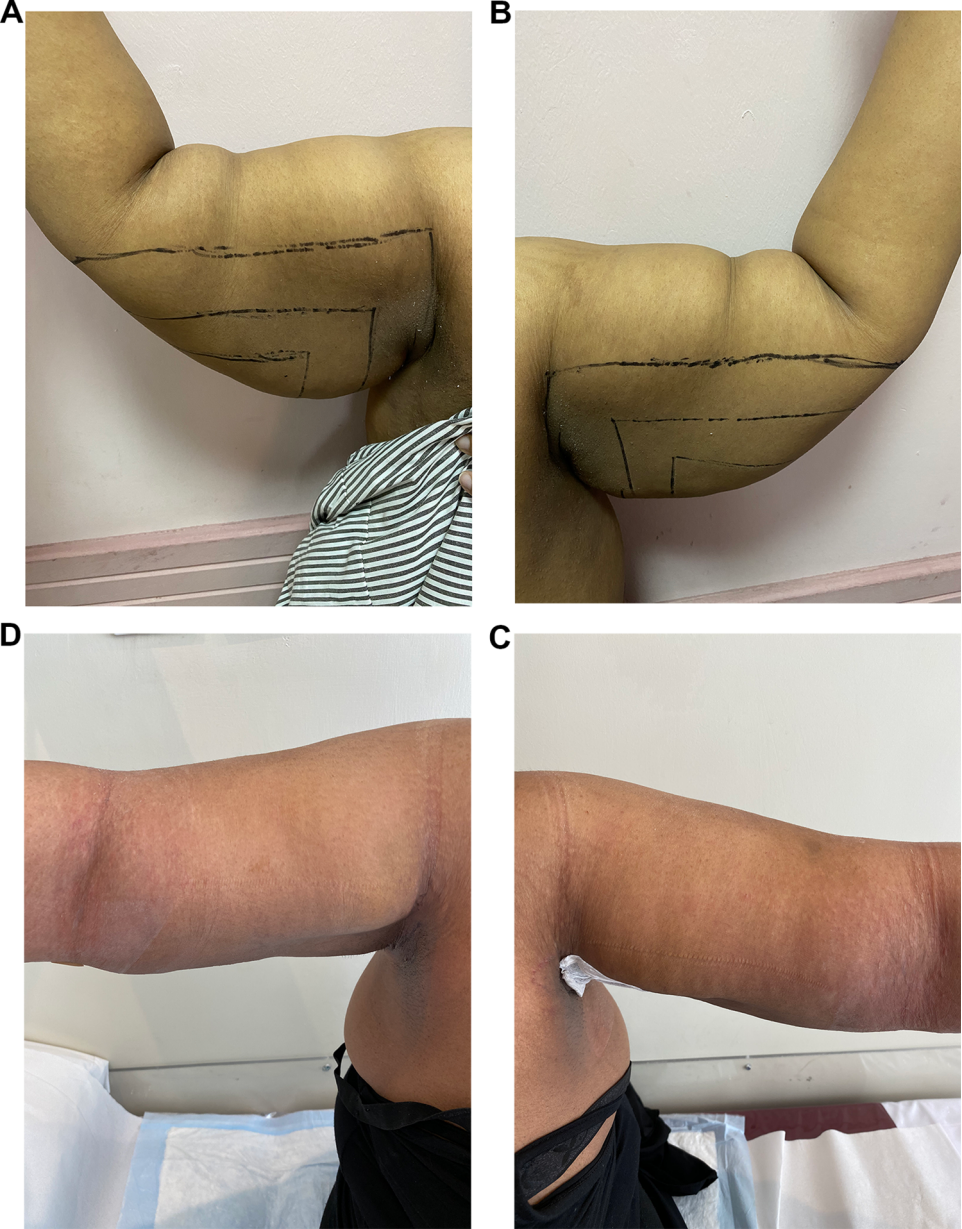
Forty-five-yo female with class IIB arm lipodystrophy underwent UAL. **A** Right arm perioperative, **B** Left arm perioperative, **C** Right arm 3 month postoperative follow-up, **D** Left arm 3 months postoperative follow-up

**Fig. 6:**
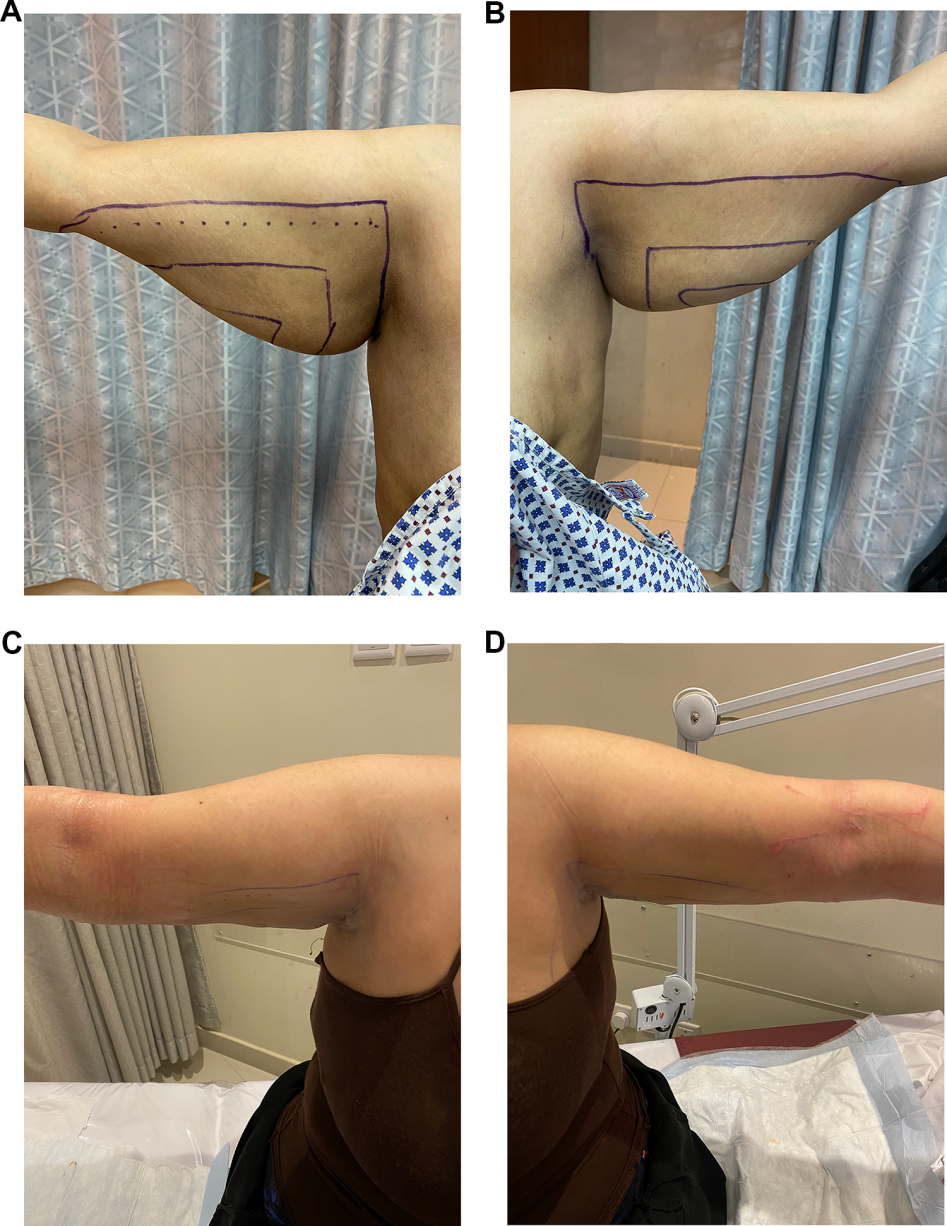
Forty-yo female with class III arm lipodystrophy underwent UAL. **A** Right arm perioperative. **B** Left arm perioperative. **C** Right arm 2 months postoperative follow-up. **D** Left arm 2 months postoperative follow-up

In the last couple of years, a dramatic evolution in arm contouring procedures had been witnessed in the published data due to an increase in the population's demand to achieve the perfect body besides the substantial awareness of losing weight [9]. Nearly those methods were just modifications in brachioplasty length, position, and design in an attempt to minimize the incision complications and improve outcome [11]. The popularization of the modern brachioplasty was conducted by Lockwood. He addressed the excision of the redundant skin through a *T*-shape design that runs through the arm and axilla [12]. Other authors described the same *T*-shape design associated with purse-string sutures to reduce the scar length [6]. More current publications included the sinusoidal pattern scar aiming to decrease the linear scar, contracture, wound dehiscence, keloid, and hypertrophic scar formation [13]. Another modification suggested placing the incision more posteriorly with a Z-plasty [14]. A fish-like design was described by Chandawarkar and Lewis [15]. Different variants of short-scar modifications had been tailored to decrease scar complications and improve the parent’s surgical outcome. L brachioplasty is with an inconspicuous scar across the axilla and chest wall [16]. For proximal deformities, Reed LS suggested a short-scar approach with only a vertical incision in the axilla [17]. Nguyen and Rohrich proposed liposuction to short-scar techniques, adopting all the merits of the short-scar brachioplasty and using liposuction for refinement of the distal part. [18] Another *S*-shaped incision is used to decrease vertical and horizontal vectors of the scar [19]. Mahfouz AI has described circumferential liposuction with brachioplasty in the same session [11]. The traditional brachioplasty is still the gold standard for post-bariatric patients (class IV) due to poor-quality skin [20]. In our practice we advised all the patient who suffers from severe laxity after any bariatric intervention with grade IV according to El-Khatib’s classification that they are not candidates for such technique. The challenge is in lipodystrophy classes II and III (El-Khatib’s classification), for which most of the literature articles are still talking about types of skin excision [10]. Rohrich RJ mentioned that, despite these modifications having made brachioplasty more safe and effective, arm numbness (from cutaneous nerve cutting) is still a common complaint regardless of the procedure used [21]. Yet, the uncharted territory in the literature is the possibility of non-excisional aesthetic refinement of the arm [9]. Circumferential liposuction only can be used for arm contouring with success, but their work was limited to early stages of lipodystrophy, and also, they experienced a 10.2% secondary procedure rate for either re-liposuction or brachioplasty [19]. After that liposuction has gained increased attention as an adjunctive procedure in brachioplasty [2]. Nguyen v Rohrich in 2010 have added UAL to their posterior brachioplasty technique and reported success apart from the upsetting scar and its limitation to specific lipodystrophy stages [18]. To date, the literature has a lack of studies that assess the versatility of liposuction alone, either SAL or energy-assisted liposuction, in managing arm lipodystrophy specifically stages II–III according to El-Khatib’s classification [10]. Most of our patients had been told by other surgeons that they are candidates for brachioplasty, and their fear of the appearance and length of the scar has led them to look for other alternatives, like that mentioned by Theodorou S in 2013 who used radiofrequency-assisted liposuction (RFAL) in contouring arm lipodystrophy [4]. In this study, we have tried to assess the effectiveness of UAL alone in arm contouring for lipodystrophy stages II–III, and the outcome was evaluated through complications incidence, patient satisfaction survey, and an independent plastic surgeon appraisal of patients' photographs. In this study, we have done 180° inferior liposuction, unlike Mahfouz and Hill et al. [11, 20] who have used circumferential liposuction and Bossert [22], who has used regional liposuction adjunctive to brachioplasty. In this study, the authors did not approach the upper region in the arm because of two main reasons; the first one is patient characteristics as most of them did not have significant lipodystrophy in that area and they did not complain of the upper part of their arm. The second reason is to avoid liposuction over the medial bicipital groove which contains the medial cutaneous nerve of the arms and forearms, basilic vein, and its branches over very deep important neurovascular structures like the medial, ulnar nerve, and brachial artery. Besides minimizing the incidence of distal swelling and lymphedema by avoiding any lymphatic disruption, the authors still believe that some cases might require conservative circumferential liposuction when the patient suffers from circumferential lipodystrophy putting into consideration the dangerous zones to minimize complications. The mean reduction in mid-arm circumference was 10.1±1.2 cm with a statistical significance between pre- and post-measurement which is higher than previously published studies [11, 23]. Our average fat aspirate volume was 1646±597 ml, which is more than that mentioned by Theodorou S (568 ml) and Mahfouz AI (588 ml) [4, 11]. The mean operative time was 1.75±0.28 h which is less than that mentioned by Theodorou S (2.03 h), who has also reported 2 (5.4%) complications (one burn and one seroma) after the use of RFAL in the arm contouring [4]. The authors have not met any complications either seroma and skin burn or other many complications reported in many published studies, [18, 20] and this may be due to the non-closure of the ports that help drainage of any collections, use of UAL power of not more than 70%, and good compression of the arm by pressure garment for 6–8 weeks. The survey results showed that 85.71 % were very satisfied and 14.29 % were satisfied, while no reported any unsatisfaction, unlike Theodorou S who reported 13% dissatisfaction, and Mahfouz AI who reported 4.8% dissatisfaction [4, 11]. The independent plastic surgeon evaluation of the patients’ photographs revealed that 92.86% of the results were excellent and 7.14 % were very good, while Theodorou S reported 8 % excellent, 72 % good, and 18 % moderate, and 2% poor [4]. Some cases (Figs. 3, 4, 5 and 6).

Although this study has some limitations regarding being a retrospective one, a small group size of the patients is in the female category with limited follow-up. Our result compared to the available literature is promising and aims to attract the attention that some cases of lipodystrophy and arm laxity could beneficially achieve excellent outcomes using liposuction advanced technology without the rush of brachioplasty that is the only way to achieve arm contouring. We suggest a prospective randomized study and long-term follow-up on a large group with a multicenter study.

## Conclusion

In this study, the authors presented a case series that demonstrated how versatile is the UAL in contouring a wide spectrum of arm lipodystrophy stages in non-post-bariatric patients which have traditionally been managed with brachioplasty with its multiple complications and the resultant extensive scar. As arm lipodystrophy patients have usually been worried about the length and appearance of brachioplasty scar, UAL presents a non-excisional alternative for arm aesthetic refinement without a rush for brachioplasty with its unpleasant complications.
